# Effect of antibiotic monensin on cell proliferation and IGF1R signaling pathway in human colorectal cancer cells

**DOI:** 10.1080/07853890.2023.2166980

**Published:** 2023-03-09

**Authors:** Youping Zhou, Youlin Deng, Jing Wang, Zhengjian Yan, Qiang Wei, Jixing Ye, Junhui Zhang, Tong-Chuan He, Min Qiao

**Affiliations:** a Department of Gastroenterology, The First Affiliated Hospital of Chongqing Medical University, Chongqing, China; b Molecular Oncology Laboratory, Department of Orthopaedic Surgery and Rehabilitation Medicine, The University of Chicago Medical Center, Chicago, IL, USA

**Keywords:** Colorectal cancer, monensin, cell proliferation, IGF signaling, cancer therapy

## Abstract

**Background/Aims:**

Colorectal cancer is the third leading cause of death in patients with cancers in America. Monensin has represented anti-cancer effect on various human cancer cells. We seek to investigate the effect of monensin on proliferation of human colorectal cancer cells and explore whether IGF1R signaling pathway is involved in anti-cancer mechanism of monensin.

**Methods:**

Cell proliferation and migration were assessed by crystal violet staining and cell wounding assay respectively. Cell apoptosis was analyzed by Hoechst 33258 staining and flow cytometry. Cell cycle progression was detected with the use of flow cytometry. Cancer-associated pathways were assessed with the use of pathway-specific reporters. Gene expression was detected by touchdown-quantitative real-time PCR. Inhibition of IGF1R was tested by immunofluorescence staining. Inhibition of IGF1R signaling was accomplished by adenovirus-mediated expression of IGF1.

**Results:**

We found that monensin not only effectively inhibited cell proliferation, cell migration as well as cell cycle progression, but also induced apoptosis and G1 arrest in human colorectal cancer cells. Monensin was shown to target multiple cancer-related signaling pathways such as Elk1, AP1, as well as Myc/max, and suppressed IGF1R expression *via* increasing IGF1 in colorectal cancer cells.

**Conclusion:**

Monensin could suppressed IGF1R expression *via* increasing IGF1 in colorectal cancer cells. It has the potential to be repurposed as an anti-colorectal cancer agent, but further studies are still required to investigate the detailed mechanisms of monensin underlying its anti-cancer motion.

**Key Messages**
- Monensin inhibits the cell proliferation and the migration, induces apoptosis and inhibits cell cycle progression in human colorectal cancer cells.
- Monensin may exert anti-cancer activity by targeting multiple signaling pathways, including the IGF1R signaling pathway.
- Monensin has the potential to be repurposed as an anti-colorectal cancer agent.

## 1. Introduction

Colorectal cancer (CRC), accounting for 8% of estimated newly diagnosed cancers in both genders, is the third leading cause of death in patients with cancers in America [1]. The 5-year survival rate in patients with CRC is strongly related to its stage at diagnosis, and is particularly poor at stage IV [1–3]. This results from the limitations of chemotherapy because of drug resistance and organ toxicities [4,5]. Therefore, it is of great urgency to develop novel therapeutics. An effective alternative to development of novel therapeutics is repurposing drugs. Nowadays, several examples of such drugs are in various stages of clinical trials [6,7].

Monensin, a polyether ionophore antibiotic secreted by the bacteria Streptomyces cinnamonensis, has represented a positive safety profile in veterinary medicine [8]. A recent study found that malignant cell lines are more than 20-fold more sensitive to monensin than their nonmalignant counterparts [9], suggesting that monensin may target cancer cells more preferentially than most conventionally-used cytotoxic chemotherapy drugs. Furthermore, monensin has exhibited cytotoxic effect against several tumor cells, including colorectal cancer cells [9–20]. Given the safety and anti-cancer effect that monensin showed, monensin has the potential to be repurposed as an anti-tumor drug. However, the mechanisms underlying the anti-cancer activities of monensin is not fully understood. Thus, we investigated the anti-cancer activity of monensin against RKO and HCT-116, two human colorectal cancer cells lines, in terms of cell proliferation, migration, apoptosis as well as cell cycle, and explored the signaling pathways probably-modulated by monensin in present study.

## 2. Materials and methods

### 2.1. Cell culture and chemicals

Human colorectal cancer cell lines RKO and HCT-116 were generously provided by Dr. Ernest Lengyel. These cells were maintained in complete Dulbecco’s Modified Eagle’s Medium (DMEM) containing 10% fetal bovine serum (FBS, Invitrogen, Garlsbad, CA), 100 units of penicillin and 100ug of streptomycin and cultured at 37 °C in a humidified atmosphere with 5% CO_2_. Chemicals monensin (aka, rumensin) were purchased from Cayman Chemical (Ann Arbor, MI). Unless indicated otherwise, all chemicals were purchased from Sigma-Aldrich (St. Louis, MO).

### 2.2. Crystal violet cell viability assay

Crystal violet staining assay was performed as described [21–23]. Briefly, sub-confluent RKO and HCT-116 cells were treated with varied concentration of monensin. At the indicated time after treatment, cells were washed with PBS and stained with 0.5% crystal violet/formalin solution at room temperature for 20-30 min. The stained cells were washed with tape water and air dried for taking macrographic images [24,25]. Then, the stained cells were dissolved in 10% acetic acid at room temperature for 20 min with shaking and measured absorbance at 570-590nm for quantitative measurement.

### 2.3. Cell wounding/migration assay

Cell wounding/migration assay was conducted as described [26,27]. Briefly, exponentially growing RKO and HCT116 cells were seeded in 6-well cell culture plates and allowed to reach approximately 90% confluence. Then the monolayer cells were wounded with sterile micro-pipette tips. At various time points, the wound healing status at the approximately same locations was recorded under bright field microscopy. Each assay condition was done in triplicate.

### 2.4. Apoptosis analysis (hoechst 33258 staining)

Hoechst 33258 staining for apoptosis analysis was performed as previously described [24,28]. Exponentially growing RKO and HCT116 cells were treated with varied concentrations of monensin or DMSO control. At 24 h post treatment, cells were collected, fixed and stained with the Magic Solution (10× stock: 0.5% NP-40, 3.4% formaldehyde, 10ug/ml Hoechst 33258, in PBS). Apoptotic cells were examined and recorded under a fluorescence microscope. Each assay condition was done in triplicate. The results were repeated at least in three independent batches of experiments. The average numbers of apoptotic cells were calculated by counting apparent apoptotic cells in at least ten random fields at 100× magnification for each assay condition.

### 2.5. Apoptosis analysis (annexin V-FITC flow cytometry)

The annexin V staining apoptosis assay was conducted as previously described [24,26,27]. Briefly, exponentially growing RKO and HCT116 cells were seeded in 6-well plates and treated with varied concentrations of monensin. At 24 h post treatment, cells were trypsinized, washed with PBS, resuspended in Annexin V Binding Buffer at a density of 10^6^ cells/ml, and stained with Annexin V-FITC (BD Pharmingen, San Jose, CA) as well as propidium iodide for 15 min at room temperature under a light –proof condition. The stained cells were subjected to flow cytometry analysis with the use of the BD FACScalibur-HTS. The acquired flow cytometry data were analyzed by using the FlowJo v10.0 software. Each assay condition was done in triplicate.

### 2.6. Cell cycle analysis

The exponentially growing RKO and HCT116 cells were seeded in 6-well plates at sub-confluence and treated with 4 micromole monensin or DMSO control. At 48 h post treatment, cells were collected, fixed and stained with the Magic Solution for 30 min. The stained cells were subjected to flow cytometry analysis with the use of the BD FACScalibur-HTS. The acquired flow cytometry data were analyzed by using the FlowJo v10.0 software. Each assay condition was done in triplicate.

### 2.7. Construction and amplification of recombinant adenovirus expressing IGF1 or RFP

Recombinant adenovirus expressing IGF1 was constructed by using the AdEasy system as described [29–32]. Briefly, the human IGF1 coding region was PCR amplified and subcloned into an adenoviral shuttle vector, and used to generate and amplify recombinant adenovirus in HCT-116 cells. The resulting adenovirus was designated as AdR-IGF1, which also expresses RFP [33–36]. Analogous adenovirus expressing only RFP (AdRFP) was used as a control [37,38]. For all adenoviral infections, polybrene (4-8µg/ml) was added to enhance infection efficiency as previously reported [39].

### 2.8. Immunofluorescence staining

The immunofluorescence staining assays were carried out as previously described [27,28]. Briefly, for IGF1 staining assay, the HCT-116 cells were first infected with AdR-IGF1 or AdRFP for 16 h, replated into 24-well plates, and then treated with monensin at varied concentrations or vehicle control. For IGF-1R staining assay, sub-confluent HCT-116 cells were treated with monensin at varied concentrations or vehicle control. At 36 h post treatment, the cells were fixed and subjected to immunofluorescence staining with antibody against IGF1 (Santa Cruz Biotechnology, Santa Cruz, CA), or IGF-1R (Santa Cruz Biotechnology). Control IgG and minus primary antibodies were used as negative controls.

### 2.9. Cell transfection and pathway-specific luciferase reporter assay

The Gaussia luciferase (GLuc) reporter assay was performed as described [40,41]. The 12 cancer-related signaling pathway GLuc reporters were homemade and previously described [40], containing NFAT, HIF1, TCF, E2F, Elk1, Ap1, NF-κB, Smad, STAT1/2, RBP-Jκ, CREB and Myc/Max reporters. A constitutively active reporter pG2Luc was used as a control [10,28]. Sub-confluent RKO cells were seeded in 25cm2 culture flasks and transfected with 3.0 µg per flask of the 13 reporter plasmids by using Lipofectamine (Invitrogen). At 16 h post transfection, cells were replated in 12-well plates and treated with varied concentrations of monensin or DMSO control. At 48 h post-treatment, culture media were taken and subjected to Gaussia luciferase assay using the BioLux Gaussia Luciferase Assay Kit (New England Biolabs). Each assay condition was done in triplicate. Luciferase activity was normalized by total cellular protein concentrations among the samples.

### 2.10. Total RNA isolation and touchdown-quantitative real-time PCR (TqPCR) analysis

Sub-confluent RKO cells were treated with varied concentrations of monesin for 48 h. Total RNA was isolated from the treated cells by using TRIZOL Reagents (Invitrogen) and subjected to reverse transcription reactions with hexamer and M-MuLV reverse transcriptase (New England Biolabs, Ipswich, MA). Such cDNA products were used as PCR templates. The qPCR primers were designed by using Primer3 program [42] and used to amplify the genes of interest. The TqPCR were carried out by using the SYBR Green-based qPCR analysis on a CFX-Connect unit (Bio-Rad Laboratories, Hercules, CA), as described [43]. The qPCR reactions were done in triplicate. GAPDH was used as a reference gene.

### 2.11. Statistical analysis

The quantitative assays were performed in triplicate and/or repeated three times. Statistical analysis was carried out using Microsoft Excel program. Data were expressed as mean ± SD. Statistical significances were determined by one-way analysis of variance and the student’s t test. A value of *p* < 0.05 was considered statistically significant.

## 3. Results

### 3.1. Monensin inhibits the cell proliferation and the migration in human colorectal cancer cells

**Figure 1.**
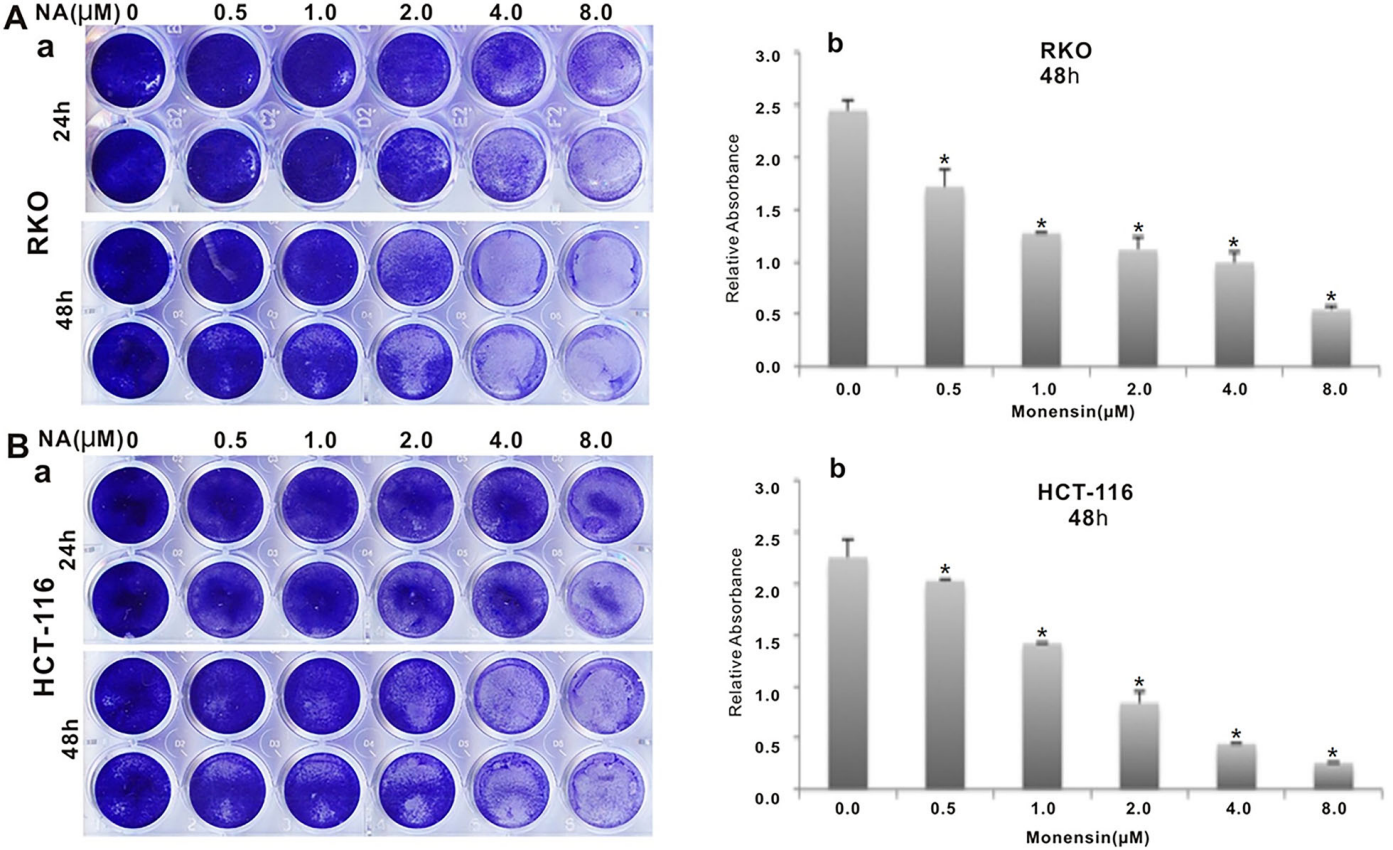
Monensin inhibits the proliferation in human colorectal cancer cells. (A) Crystal violet staining assay for RKO cells treated with increasing concentrations in two indicated times. Monensin effectively inhibits the proliferative activity of RKO cell in a dose-dependent and time-dependent manner (a). The optical absorbance for crystal violet staining assay at 48 h post-treatment significantly declines with increasing concentrations of monensin in RKO cell (*, *p* < 0.*05*) (b). (B) Crystal violet staining assay for HCT-116 cells treated with increasing concentrations in three indicated times. Monensin effectively inhibits the proliferative activity of HCT-116 cell in a dose-dependent and time-dependent manner (a). The optical absorbance for crystal violet staining assay at 48 h post-treatment significantly declines with increasing concentrations of monensin in HCT-116 cell (*, *p* < 0.*05*) (b). Each assay condition was done in triplicate.

**Figure 2.**
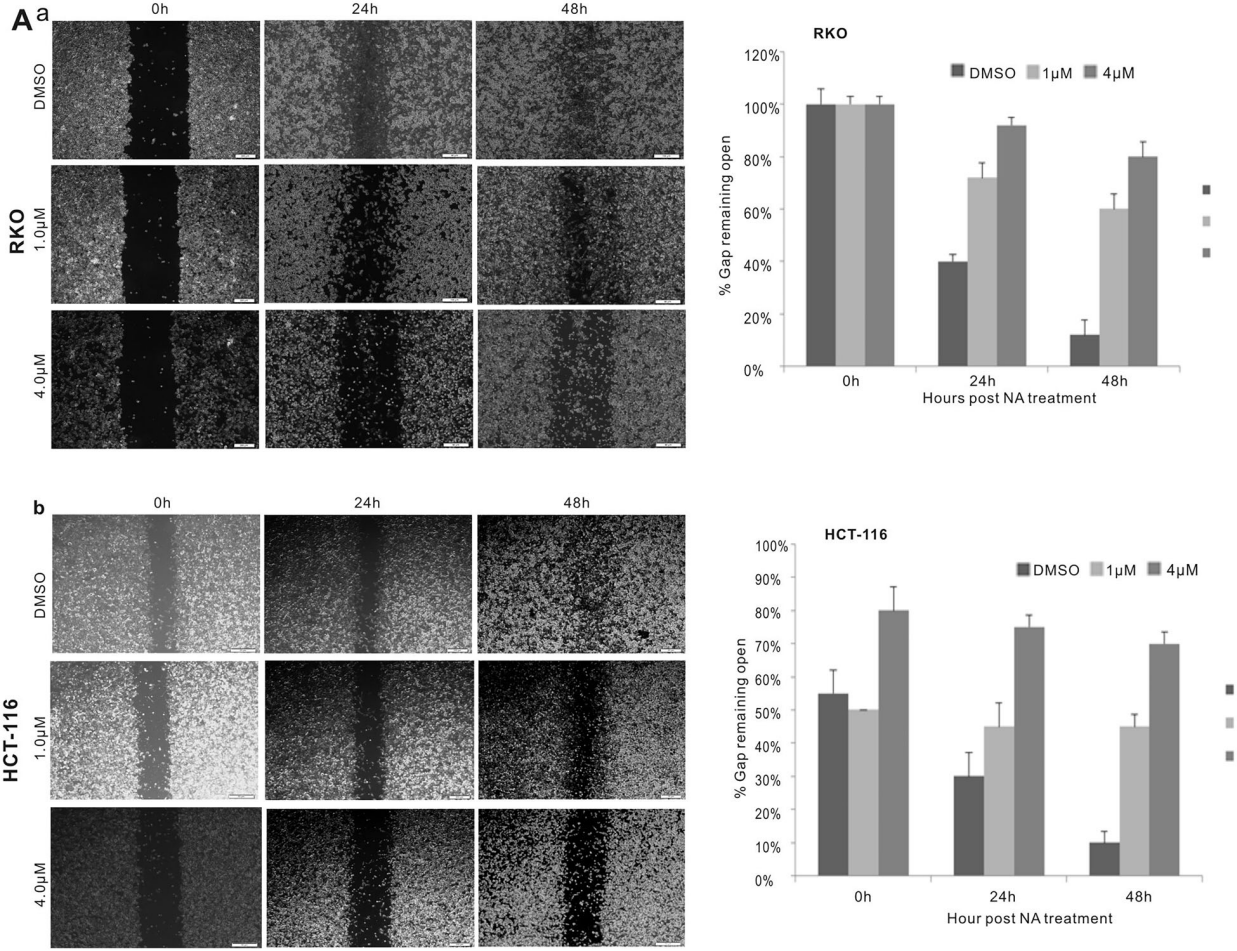
Monensin inhibits the cell migration in human colorectal cancer cells. (a) Cell wounding/migration assay for RKO cells treated with indicated concentration of monensin after the freshly confluent monolayer cells were wounded. (b) Cell wounding/migration assay for HCT-116 cells treated with indicated concentration of monensin after the freshly confluent monolayer cells were wounded. Each assay condition was done in triplicate.

To investigate the effect of the antibiotic monensin on the proliferative activity of two commonly-used human colorectal cancer cell lines RKO and HCT-116, the two sub-confluent cells were treated with increasing concentrations of monesin from 0 µM to 8 µM. Crystal violet staining results at two different post-treatment times (24 h and 48 h) showed that monensin effectively inhibited the cell proliferative activity in both RKO and HCT-116 cell lines in a dose-dependent and time-dependent manner (Figure 1(A,B), panel a). It was confirmed by quantitative analysis of crystal violet staining data (*p* < 0.05 for three indicated post-treatment times (Figure 1(A,B), panel b). Next, we conducted the cell wounding assay to test whether monensin exerts any effect on cell migration in human colorectal cancer cells. Freshly confluent RKO and HCT-116 monolayer cells were wounded and treated with 0, 0.5, 1, 2, 4 or 8 µM monensin. The wound healing status at the approximately same locations was recorded at 0 h, 24 h and 48 h after monensin treatment. The decreases of gap closure in both cell lines with 1 or 4 µM monensin at the indicated post-treatment time are presented in Figure 2 when compared to the corresponding DMSO control groups. Especially, a remarkable decrease of gap closure was observed in HCT-116 when the cells were exposed to 4 µM monensin (Figure 2(b)). In summary, the above results suggest that monensin inhibits the cell proliferation and the migration of human colorectal cancer cells.

### 3.2. Monensin induces apoptosis and inhibits cell cycle progression in human colorectal cancer cells

**Figure 3.**
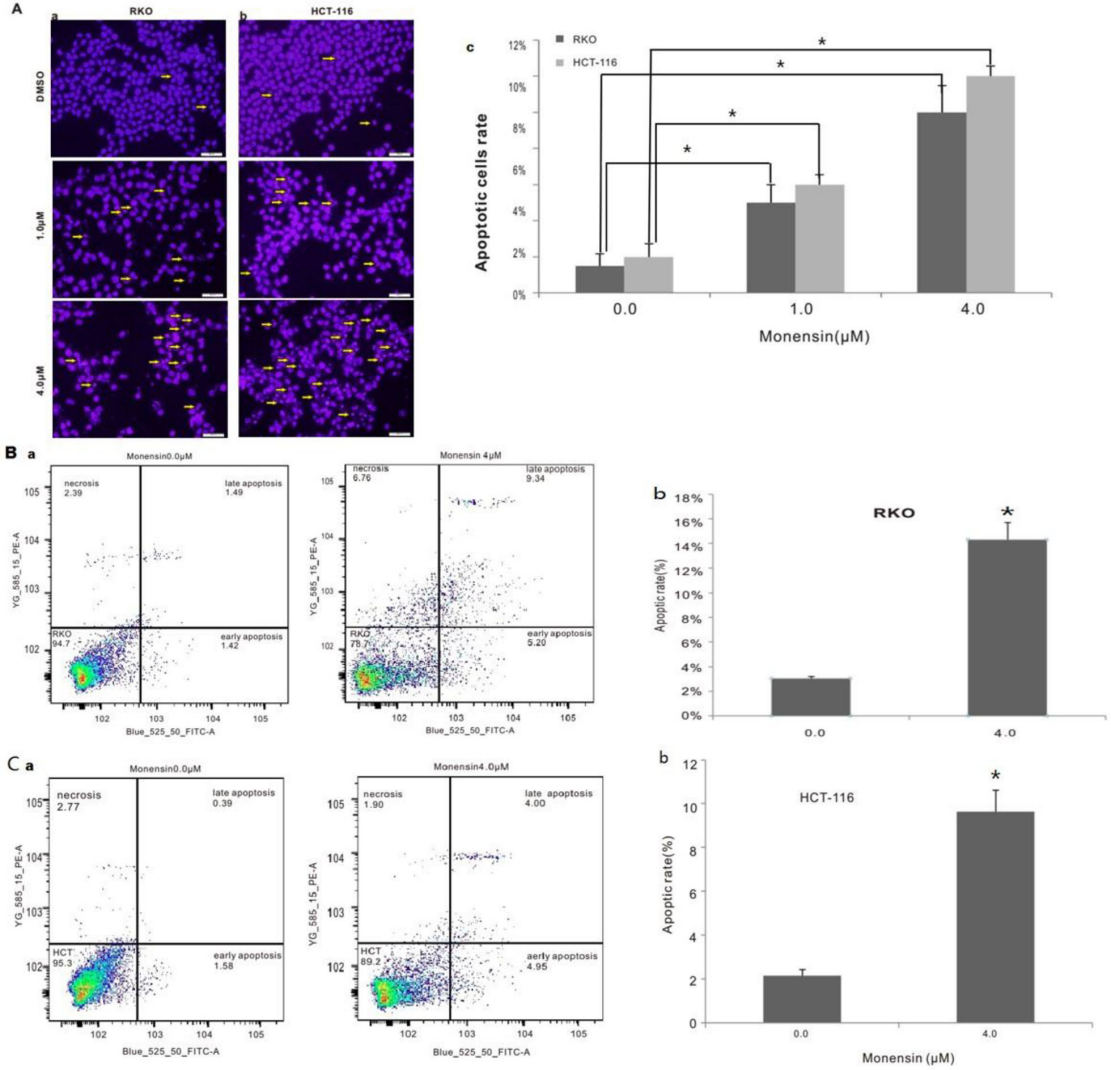
Monensin induces apoptosis in human colorectal cancer cells. (A) Hoechst 33258 staining assay for RKO (a) and HCT-116 (b) treated with varied concertrations of monensin at 24 h post-treatment. Apparent apoptotic cells were counted in at least 10 random felds under 100× magnification. The apoptotic rate rose significantly in monensin-treated RKO and HCT-116 cells (*, *p* < 0.*05*) (c). (B) Annexin V-FITC flow cytometry analysis for RKO (a). (b)*, *p* < 0.*05* (monensin-treated group vs. control group). (C) Annexin V-FITC flow cytometry analysis for HCT-116 (a). (b)*, *p* < 0.*05* (monensin-treated group vs. control group). Each assay condition was done in triplicate.

**Figure 4.**
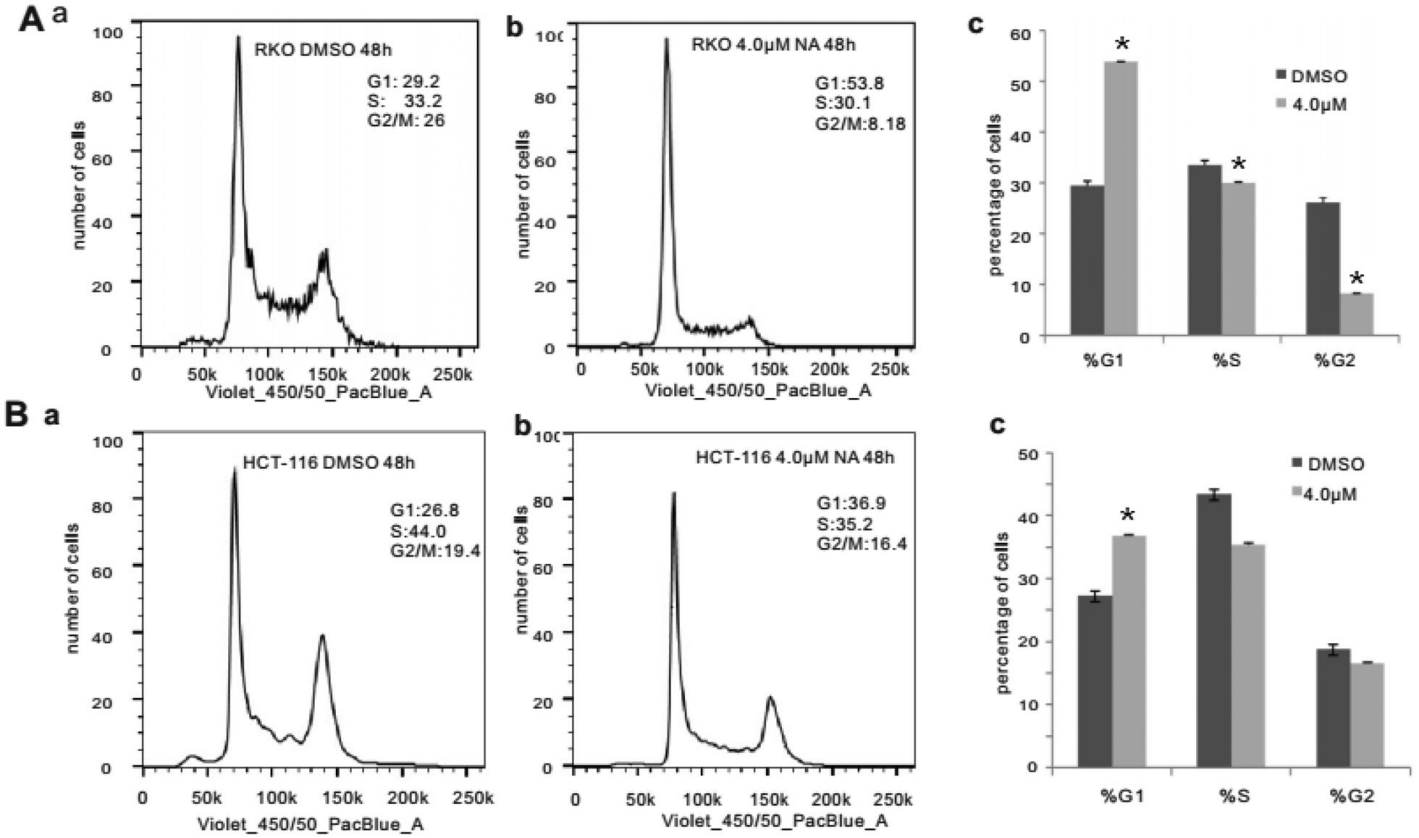
Monensin induces cell cycle progression in human colorectal cancer cells. (A) Cell cycle analysis for RKO in monensin-treated group (a) as well as control group (b). (c)*, *p* < 0.*05* (monensin-treated group vs. control group). (B) Cell cycle analysis for HCT-116 in monensin-treated group (a) as well as control group (b). (c)*, *p* < 0.*05* (monensin-treated group vs. control group). Each assay condition was done in triplicate.

Subsequently, we conducted apoptosis analysis with Hoechst 33258 staining as well as Annexin V-FITC flow cytometry to investigate whether monensin inhibits the cell proliferation in human colorectal cancer cells *via* cell apoptosis in part. Interestingly, the Hoechst 33258 staining results demonstrated that the number of apoptotic cells and the apoptotic rate rose significantly in the treatment groups with 1 µM or 4 µM concentration of monensin when compared to the 0 µM ones in both RKO and HCT-116 cell lines (*p* < 0.05, Figure 3(A)). Simultaneously, it was confirmed that the apoptotic rate significantly increased in monensin-treated RKO and CHT-116 cells by quantitative analysis of flow cytometry (*p* < 0.05, Figure 3(B,C)). In addition to apoptosis, the cell cycle between monensin-treated group and control group was also investigated. A significant increase in cell arrested in G1 phrase was found in both monensin-treated RKO and HCT-116 cells (*p* < 0.05, Figure 4(A), panel c and Figure 4(B) panel c). However, the significantly decreased cells in S/M phase was only observed in monensin-treated RKO (*p* < 0.05, Figure 4(A), panel c). These results suggest that monensin-induced apoptosis and cell cycle arrest may contribute in part to the monensin inhibition of cell proliferation in human colorectal cancer cells.

### 3.3. Monensin inhibits multiple cancer-related signaling pathways, including the downstream effectors of IGF signaling

**Figure 5.**
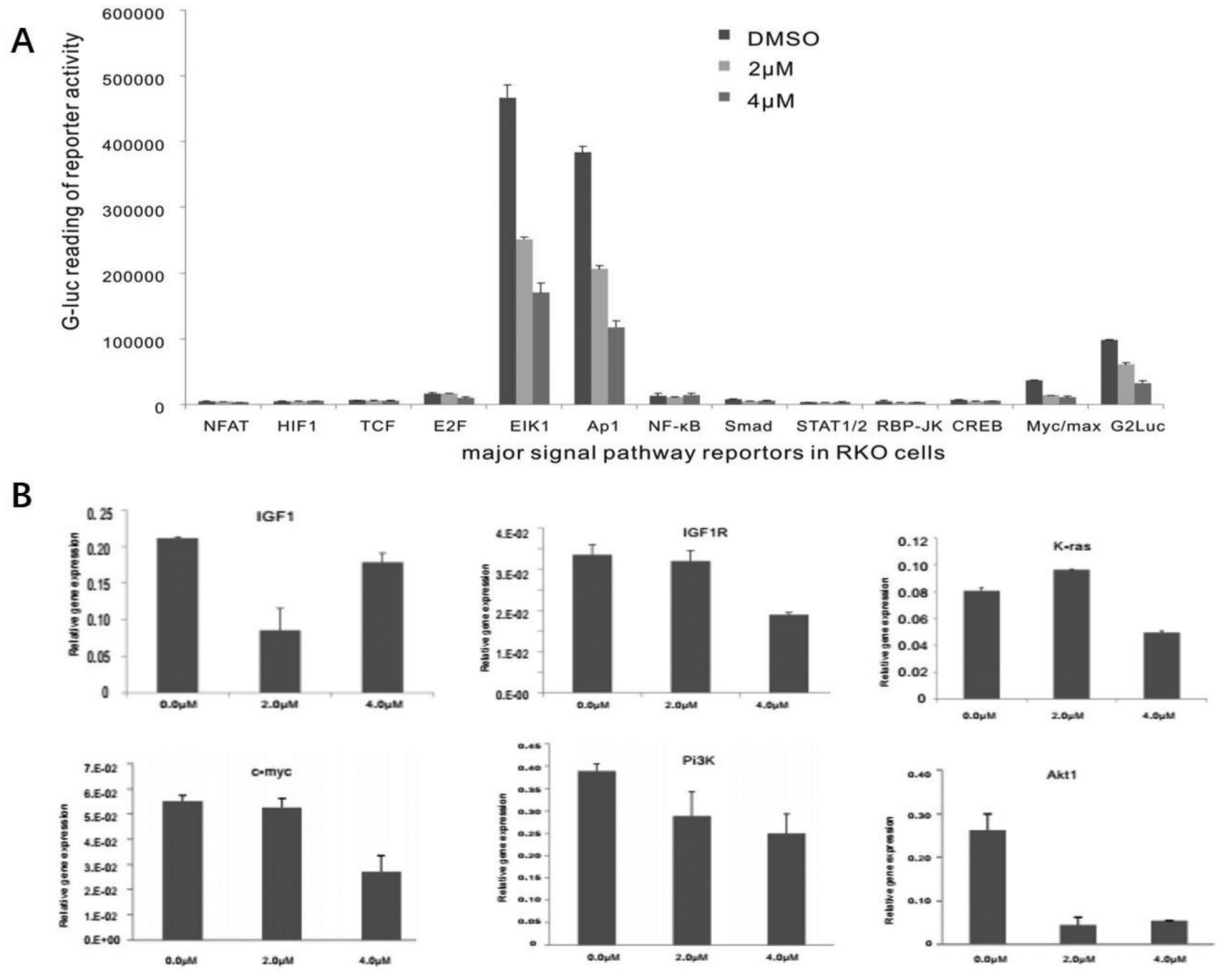
Monensin inhibits multiple cancer-related signaling pathways, including the downstream effectors of IGF signaling. (A) Effect of monensin on the 12 cancer-related pathway reporters pathways. ElK1, Ap1 and Myc/max were significantly attenuated in RKO cells exposed to either 2 µM or 4 µM of monensin at 48 h post-treatment (*, *p* < 0.*05*). (B) 4 µM concentration of Monensin effectively inhibited the expression of genes, including K-ras, c-myc, Pi3K and Akt1.But no dose-dependent fashion in monensin-induced inhibition was showed. The augment in K-ras expression, instead of inhibition, was found in group with 2 µM of monensin, while the inhibition to Akt1 was stronger in group with 2 µM of monensin than in group with 4 µM of monensin. An ascending tendency was showed in the expression of IGF1 when the cells treated with increasing concentrations of monensin, whereas a descending tendency was found in the expression of IGF1R. Each assay condition was done in triplicate.

It was reported that monensin can attenuate aberrant Wnt signaling in human colorectal cancer cell SW480 to inhibit the tumor progression in a recent study [12]. And our previous study revealed that monensin can inhibit multiple cancer-associated signaling pathways, including EGFR-signaling, in human ovarian cancer cells [10]. We asked whether other signaling pathways are involved in the suppressive effect of monensin on human colorectal cancer cells. By introducing a panel GLuc reporters for 12 cancer-associated pathways as described [40,41], along with a constitutively active reporter pG2Luc, we found that three pathways, containing ElK1, Ap1 and Myc/max, were significantly attenuated in RKO cells exposed to either 2 µM or 4 µM of monensin at 48 h post-treatment (*p* < 0.05, Figure 5(A)). The inhibitory effect of monensin on ElK1, Ap1 and Myc/max in RKO cells was markedly exhibited in 4 µM, which indicated that the inhibition was dose-dependent. Given that the reporter assays suggested that monensin may target the growth factor signaling pathways, we evaluated expression levels of the proliferation associated genes following the monensin treatment. Using our recently optimized touchdown-quantitative real-time PCR or TqPCR [43], we found K-ras, c-myc, PI3K and Akt1 was effectively decreased when the RKO cells were treated with 4 µM of monensin at 48 h post-treatment (Figure 5(B)). But no dose-dependent fashion was observed. The augment in K-ras expression, instead of inhibition, was found in group with 2 µM of monensin, while the inhibition to Akt1 was stronger in group with 2 µM of monensin than in group with 4 µM of monensin (Figure 5(B)). When the cells were treated with both 2 and 4 µM of monensin, the expression of IGF1 were lower than that of the control group, whereas a descending tendency was found in the expression of IGF1R (Figure 5(B)). It is suggested that monensin may influence expression of IGF1 and IGF1R.

### 3.4. Monensin effectively inhibits the expression of IGF1R, and may suppress the proliferation of human colorectal cancer cells *via* IGF1R inhibition

**Figure 6.**
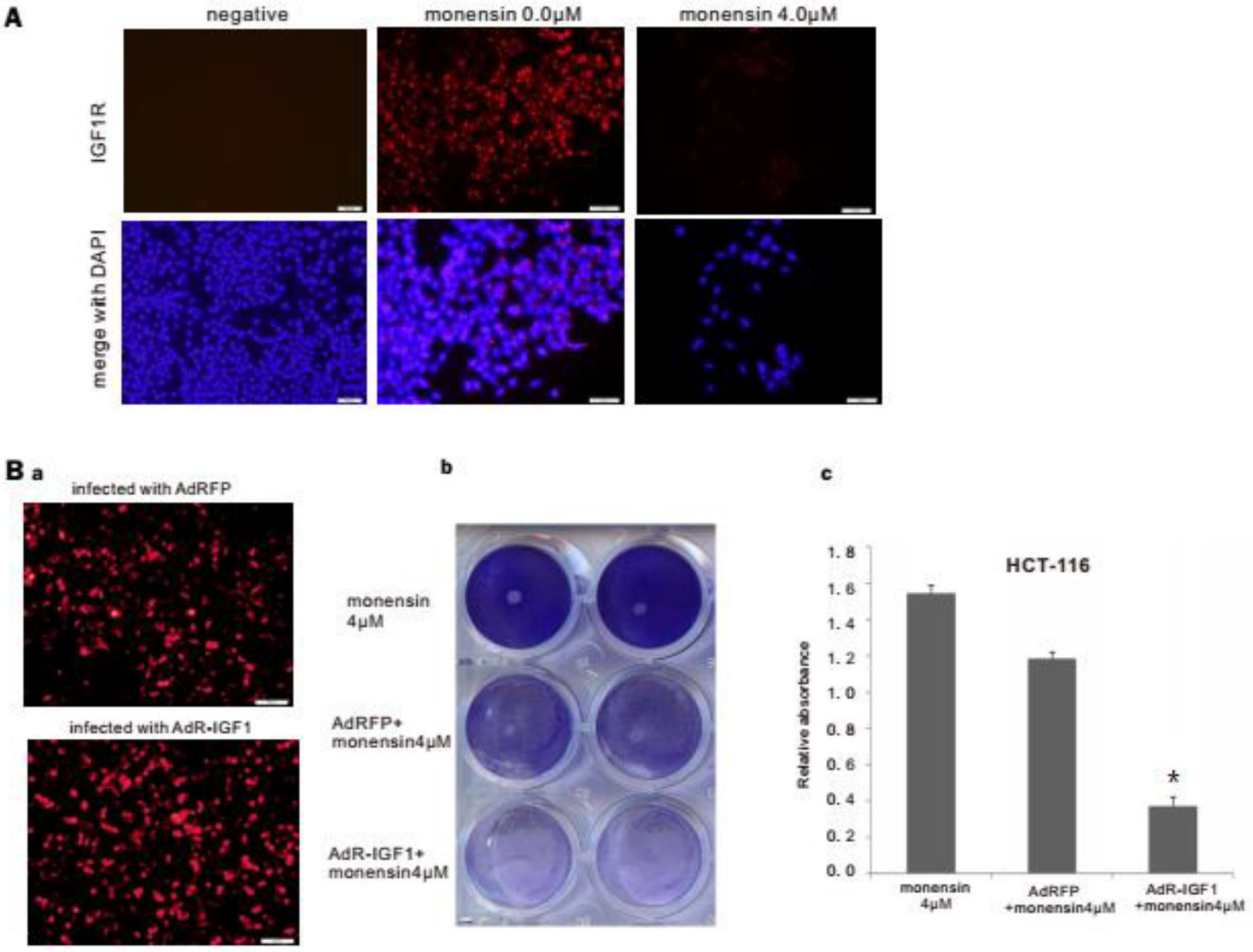
Monensin effectively inhibits the expression of IGF1R, and may suppress the proliferation of human colorectal cancer cells *via* IGF1R inhibition. (A) Immunofluorescence staining for IGF1R in HCT-116. Monensin effectively inhibited the expression of IGF1R in HCT-116 cells. (B) Immunofluorescence staining assay and crystal violet staining assessment. (a) Immunofluorescence staining for AdRFP and AdR-IGF1. (b) The crystal violet staining at 48 h post-treatment demonstrated that transfection with AdR-IGF1 effectively amplified the inhibitory effect of 4 µM of monensin on cell proliferation in human colorectal cancer cells HCT-116. (c) The optical absorbance for crystal violet staining assay at 48 h post-treatment significantly declines with when the HCT-116 cells were monensin-treated with transfection of AdR-IGF1 (*p < 0.*05*). Each assay condition was done in triplicate.

In order to demonstrate the inhibitory effect of monensin on the expression of IGF1R, which was found in Tq-PCR, we conducted the immunofluorescence staining for IGF1R post treatment with 4 µM of monensin. The results of immunofluorescence staining revealed an effective suppression on IGF1R expression in HCT-116 treated with 4 µM of monensin (Figure 6(A)). Given that the TqPCR assay suggested that monensin may reduce IGF1R *via* elevating expression of IGF1, we constructed the recombinant adenovirus AdR-IGF1, which expresses not only IGF1 but also RFP. Analogous adenovirus expressing only RFP (AdRFP) was used as a control (Figure 6(B), panel a). The crystal violet staining at 48 h post-treatment demonstrated that transfection with AdR-IGF1 effectively amplified the inhibitory effect of 4 µM of monensin on cell proliferation in human colorectal cancer cells HCT-116 (Figure 6(B), panel b). It was confirmed by quantitative analysis of crystal violet staining data (*p* < 0.05, Figure 6(B), panel c.). It is suggested that monensin can down-regulate the expression of IGF1R in human colorectal cancer cells, which has a synergistic anti-proliferation effect with overexpression of IGF1.

## 4. Discussions

### 4.1. Monensin may be repurposed as an effective anti-cancer agent for human tumors, including colorectal cancer

The patients with colorectal cancer at stage IV accounts for about 20-25% of total patients, whose 5-year survival rate only reaches about 10% and much less than that at stage I-II, which ranges from 80% to 90% [1,3]. This is due to the limitations of chemotherapy resulting from drug resistance and organ toxicities [4,5]. Thus, searching and developing a novel effective therapeutic agency become critical to treat colorectal cancer. Various ionophore antibiotics, including monensin, salinomycin and nigericin, have been reported to overcome multidrug resistance in different types of cancer cells [44,45], Present study showed that monensin effectively inhibited cell proliferation as well as migration, and induced apoptosis as well as cell cycle arrest in RKO and HCT-116. Similar results have been reported in other researches with respect to various kinds of cancers, including prostate, ovarian, lung, glioma, renal, myeloma, colorectal cancer cell lines and so on [9–20]. Furthermore, a recent study indicated that malignant neoplasm showed more sensitive to monensin than their nonmalignant counterparts [9]. Simultaneously, as an antibiotic, monensin has represented a positive safety profile in veterinary medicine and been applied in cattle and poultry feed for nearly 50 years [8,20]. Given the features described above, monensin has the potential to be repurposed as the novel therapeutic agency against tumor, including colorectal cancer.

### 4.2. Monensin may exert anti-cancer activity by targeting multiple signaling pathways

Given that monensin possesses a positive safety profile and acts effectively at concentrations from low nanomolar to micromolar in various cancers as reported [9–20], to investigate the mechanisms of monensin underlying the anti-cancer motion is necessary. Earlier and recent studies have showed that a high ratio of Bax to Bcl-2, activation of caspase family, including caspase-3, caspase-8 and caspase-9, as well as the loss of mitochondrial transmenbrane potiential are observed in monensin-induced apoptosis of several human cancer cell lines [10,13–18]. Besides apoptosis, the arrest of cell cycle has also been reported in monensin-treated human cancer cell. G1 arrest accompanying decrease of CKD6, cyclin A and cyclin D1 have been demonstrated in most human cancer cell lines exposed to monensin [10,12–15,17,18]. Consistent with the earlier researches, present study also showed that the human colorectal cancer cells, RKO and HCT-116, were induced apoptosis and G1 arrest in cell cycle after monensin treatment. Simultaneously, monensin was indicated to be a potent inducer of oxidative stress and inhibitor of androgen signaling leading to apoptosis in prostate cancer cells [9]. Furthermore, monensin was demonstrated to induce endoplasmic reticulm stress to up-regulate DR5 and down-regulate c-FLIP in glioma cells [19]. In addition, monensin was shown to inhibit canonical Wnt signaling in human colorectal cancer cells SW480 and to suppress tumor growth in multiple intestinal neoplasia mice [12]. More recently, monensin was suggested to inhibit the growth factor receptor-induced signaling pathways and suppress EGFR expression [10]. Taken together, it is evidently suggested that monensin may target cancer cells *via* multiple mechanisms.

We analyzed the effect of monensin on 12 cancer-related pathways and found that monensin inhibited the reporter activities for ElK1, Ap1 pathway, and to a lesser extent the Myc/max reporter activity in human colorectal cancer cells. Next, the expressions of five gene, containing IGF1R, K-ras, c-myc, PI3K and Akt1 were effectively inhibited by 4uM monensin, whereas the expression of IGF1 was amplified. It has been showed that IGF1R plays a key role in the colorectal cancer [46,47] and PI3K/Akt as well as Ras/Raf/MEK/ERK pathways are the downstream mediators of the IGF1R signaling in cancers [48]. Collectively, the results in present study strongly suggest that monensin is probably to exert anti-cancer activity by inhibiting the IGF1R signaling pathway *via* IGF1 increase. The immunofluorescence staining in present study revealed that monensin effectively suppresses the IGF1R expression. Consistent with the hypothesis, monensin combined with AdR-IGF1 transfection significantly enhanced the inhibition to cell proliferation in human colorectal cancer cells. It suggests that monensin combined with treatment targeting IGF1R or IGF1 may potentiate anti-cancer effect in colorectal cancer, which needs further investigation. In addition, a recent research indicated that monensin inhibits epidermal growth factor receptor (EGFR) trafficking and activation [20]. Interestingly, our recent study also revealed that monensin effectively inhibited EGFR and tumor grow in human ovarian cancer, however no inhibition to IGF1R was observed [10]. It is suggested that the mechanism of monensin underlying anti-cancer motion may depend on the cancer cell species. But the effect of monensin on EGFR was not involved in present study. Further investigations with respect to effect of monensin on EGFR in colorectal cancer cells are required.

## 5. Conclusions

In summary, we investigated the potential of repurposing monensin as an anti-cancer agent for human colorectal cancer. Our results revealed that monensin not only effectively inhibited cell proliferation, and cell migration as well as cell cycle progression, but also induced apoptosis and G1 arrest of human colorectal cancer cells. Monensin was shown to target multiple cancer-related signaling pathways such as Elk1, AP1, as well as Myc/max, and suppressed IGF1R expression *via* increasing IGF1 in colorectal cancer cells. Thus, our results suggest that monensin has the potential to be repurposed as an anti-colorectal cancer agent. But further studies are still required to investigate the detailed mechanisms of monensin underlying its anti-cancer motion.

## Data Availability

On reasonable request to the corresponding author.

## References

[1] Siegel RL, Miller KD, Jemal A. Cancer statistics, 2016. CA: A Cancer J Clinic. 2015;66:7–30. 10.3322/caac.21332 26742998

[2] Atkin WS, Edwards R, Kralj-Hans I, UK Flexible Sigmoidoscopy Trial Investigators, et al. Once-only flexible sigmoidoscopy screening in prevention of colorectal cancer: a multicentre randomised controlled trial. Lancet. 2010;375(9726):1624–1633. 20430429 10.1016/S0140-6736(10)60551-X

[3] Brenner H, Kloor M, Pox CP. Colorectal cancer. Lancet. 2014;383(9927):1490–1502. 24225001 10.1016/S0140-6736(13)61649-9

[4] Miyamoto S, Nakanishi M, Rosenberg DW. Suppression of Colon carcinogenesis by targeting notch signaling. Carcinogenesis. 2013;34(10):2415–2423. 23729655 10.1093/carcin/bgt191 PMC3786381

[5] Suliman MA, Zhang Z, Na H, et al. Niclosamide inhibits Colon cancer progression through downregulation of the notch pathway and upregulation of the tumor suppressor miR-200 family. Int J Mol Med. 2016;38(3):776–784. 27460529 10.3892/ijmm.2016.2689 PMC4990307

[6] Gupta SC, Sung B, Prasad S, et al. Cancer drug discovery by repurposing: teaching new tricks to old dogs. Trends Pharmacol Sci. 2013;34(9):508–517. 23928289 10.1016/j.tips.2013.06.005

[7] Wang ZY, Quan Y, Zhang HY. Medical genetic inspirations for anticancer drug repurposing. Trends Pharmacol Sci. 2014;35(1):1–3. 24315157 10.1016/j.tips.2013.11.002

[8] Pressman BC. Ionophorous antibiotics as models for biological transport. Fed Proc. 1968;27(6):1283–1288. 5725218

[9] Ketola K, Vainio P, Fey V, et al. Monensin is a potent inducer of oxidative stress and inhibitor of androgen signaling leading to apoptosis in prostate cancer cells. Mol Cancer Ther. 2010;9(12):3175–3185. 21159605 10.1158/1535-7163.MCT-10-0368

[10] Deng Y, Zhang J, Wang Z, et al. Antibiotic monensin synergizes with EGFR inhibitors and oxaliplatin to suppress the proliferation of human ovarian cancer cells. Sci Rep. 2015;5:17523. 26639992 10.1038/srep17523 PMC4671000

[11] Choi HS, Jeong EH, Lee TG, et al. Autophagy inhibition with monensin enhances cell cycle arrest and apoptosis induced by mTOR or epidermal growth factor receptor inhibitors in lung cancer cells. Tuberc Respir Dis. 2013;75(1):9–17. 10.4046/trd.2013.75.1.9 PMC3741474 23946753

[12] Tumova L, Pombinho AR, Vojtechova M, et al. Monensin inhibits canonical wnt signaling in human colorectal cancer cells and suppresses tumor growth in multiple intestinal neoplasia mice. Mol Cancer Ther. 2014;13(4):812–822. 24552772 10.1158/1535-7163.MCT-13-0625

[13] Park WH, Kim ES, Jung CW, et al. Monensin-mediated growth inhibition of SNU-C1 Colon cancer cells via cell cycle arrest and apoptosis. Int J Oncol. 2003;22(2):377–382. 12527937

[14] Park WH, Jung CW, Park JO, et al. Monensin inhibits the growth of renal cell carcinoma cells via cell cycle arrest or apoptosis. Int J Oncol. 2003;22(4):855–860. 12632079

[15] Park WH, Kim ES, Kim BK, et al. Monensin-mediated growth inhibition in NCI-H929 myeloma cells via cell cycle arrest and apoptosis. Int J Oncol. 2003;23(1):197–204. 12792794

[16] Park WH, Seol JG, Kim ES, et al. Monensin-mediated growth inhibition in human lymphoma cells through cell cycle arrest and apoptosis. Br J Haematol. 2002;119(2):400–407. 12406077 10.1046/j.1365-2141.2002.03834.x

[17] Park WH, Lee MS, Park K, et al. Monensin-mediated growth inhibition in acute myelogenous leukemia cells via cell cycle arrest and apoptosis. Int J Cancer. 2002;101(3):235–242. 12209973 10.1002/ijc.10592

[18] Kim SH, Kim KY, Yu SN, et al. Monensin induces PC-3 prostate cancer cell apoptosis via ROS production and Ca2+ homeostasis disruption. Anticancer Res. 2016;36(11):5835–5843. 27793906 10.21873/anticanres.11168

[19] Yoon MJ, Kang YJ, Kim IY, et al. Monensin, a polyether ionophore antibiotic, overcomes TRAIL resistance in glioma cells via endoplasmic reticulum stress, DR5 upregulation and c-FLIP downregulation. Carcinogenesis. 2013;34(8):1918–1928. 23615398 10.1093/carcin/bgt137

[20] Dayekh K, Johnson-Obaseki S, Corsten M, et al. Monensin inhibits epidermal growth factor receptor trafficking and activation: synergistic cytotoxicity in combination with EGFR inhibitors. Mol Cancer Ther. 2014;13(11):2559–2571. 25189541 10.1158/1535-7163.MCT-13-1086

[21] Luo X, Chen J, Song WX, et al. Osteogenic BMPs promote tumor growth of human osteosarcomas that harbor differentiation defects. Lab Invest. 2008;88(12):1264–1277. 18838962 10.1038/labinvest.2008.98 PMC9901484

[22] Su Y, Luo X, He BC, et al. Establishment and characterization of a new highly metastatic human osteosarcoma cell line. Clin Exp Metastasis. 2009;26(7):599–610. 19363654 10.1007/s10585-009-9259-6

[23] Su Y, Wagner ER, Luo Q, et al. Insulin-like growth factor binding protein 5 suppresses tumor growth and metastasis of human osteosarcoma. Oncogene. 2011;30(37):3907–3917. 21460855 10.1038/onc.2011.97

[24] He BC, Gao JL, Zhang BQ, et al. He TC: tetrandrine inhibits wnt/beta-catenin signaling and suppresses tumor growth of human colorectal cancer. Mol Pharmacol. 2011;79(2):211–219. 20978119 10.1124/mol.110.068668 PMC3033706

[25] Wang N, Zhang W, Cui J, et al. The piggyBac Transposon-Mediated expression of SV40 T antigen efficiently immortalizes mouse embryonic fibroblasts (MEFs). PLoS One. 2014;9(5):e97316. 24845466 10.1371/journal.pone.0097316 PMC4028212

[26] He BC, Chen L, Zuo GW, et al. Synergistic antitumor effect of the activated PPARgamma and retinoid receptors on human osteosarcoma. Clin Cancer Res. 2010;16(8):2235–2245. 20371684 10.1158/1078-0432.CCR-09-2499

[27] Li R, Zhang W, Cui J, et al. Targeting BMP9-promoted human osteosarcoma growth by inactivation of notch signaling. Curr Cancer Drug Targets. 2014;14(3):274–285. 24605944 10.2174/1568009614666140305105805

[28] Liao Z, Nan G, Yan Z, et al. The anthelmintic drug niclosamide inhibits the proliferative activity of human osteosarcoma cells by targeting multiple signal pathways. Curr Cancer Drug Targets. 2015;15(8):726–738. 26118906 10.2174/1568009615666150629132157

[29] Cheng H, Jiang W, Phillips FM, et al. Osteogenic activity of the fourteen types of human bone morphogeneticproteins (BMPs). J Bone Joint Surg Am. 2003;85-A:1544–1552. 10.2106/00004623-200308000-00017 12925636

[30] Kang Q, Song WX, Luo Q, et al. A comprehensive analysis of the dual roles of BMPs in regulating adipogenic and osteogenic differentiation of mesenchymal progenitor cells. Stem Cells Dev. 2009;18(4):545–559. 18616389 10.1089/scd.2008.0130 PMC3132950

[31] Kang Q, Sun MH, Cheng H, et al. Characterization of the distinct orthotopic bone-forming activity of 14 BMPs using recombinant adenovirus-mediated gene delivery. Gene Ther. 2004;11(17):1312–1320. 15269709 10.1038/sj.gt.3302298

[32] Luo J, Deng ZL, Luo X, et al. A protocol for rapid generation of recombinant adenoviruses using the AdEasy system. Nat Protoc. 2007;2(5):1236–1247. 17546019 10.1038/nprot.2007.135

[33] Kong Y, Zhang H, Chen X, et al. Destabilization of heterologous proteins mediated by the GSK3beta phosphorylation domain of the beta-Catenin protein. Cell Physiol Biochem. 2013;32(5):1187–1199. 24335169 10.1159/000354518 PMC4064945

[34] Liu X, Qin J, Luo Q, et al. Cross-talk between EGF and BMP9 signalling pathways regulates the osteogenic differentiation of mesenchymal stem cells. J Cell Mol Med. 2013;17(9):1160–1172. 23844832 10.1111/jcmm.12097 PMC4118175

[35] Wang Y, Hong S, Li M, et al. Noggin resistance contributes to the potent osteogenic capability of BMP9 in mesenchymal stem cells. J Orthop Res. 2013;31(11):1796–1803. 23861103 10.1002/jor.22427

[36] Gao Y, Huang E, Zhang H, et al. Crosstalk between wnt/beta-Catenin and estrogen receptor signaling synergistically promotes osteogenic differentiation of mesenchymal progenitor cells. PLoS One. 2013;8(12):e82436. 24340027 10.1371/journal.pone.0082436 PMC3855436

[37] Tang N, Song WX, Luo J, et al. He TC: BMP9-induced osteogenic differentiation of mesenchymal progenitors requires functional canonical wnt/beta-catenin signaling. J Cell Mol Med. 2009;13(8B):2448–2464. 19175684 10.1111/j.1582-4934.2008.00569.x PMC4940786

[38] Ye J, Wang J, Zhu Y, et al. A thermoresponsive polydiolcitrate-gelatin scaffold and delivery system mediates effective bone formation from BMP9-transduced mesenchymal stem cells. Biomed Mater. 2016;11(2):025021. 27097687 10.1088/1748-6041/11/2/025021

[39] Zhao C, Wu N, Deng F, et al. Adenovirus-mediated gene transfer in mesenchymal stem cells can be significantly enhanced by the cationic polymer polybrene. PLoS One. 2014;9(3):e92908. 24658746 10.1371/journal.pone.0092908 PMC3962475

[40] Gao JL, Lv GY, He BC, et al. Ginseng saponin metabolite 20(S)-protopanaxadiol inhibits tumor growth by targeting multiple cancer signaling pathways. Oncol Rep. 2013;30(1):292–298. 23633038 10.3892/or.2013.2438 PMC3729206

[41] Li M, Chen Y, Bi Y, et al. Establishment and characterization of the reversibly immortalized mouse fetal heart progenitors. Int J Med Sci. 2013;10(8):1035–1046. 23801891 10.7150/ijms.6639 PMC3691803

[42] Untergasser A, Cutcutache I, Koressaar T, et al. Primer3–new capabilities and interfaces. Nucleic Acids Res. 2012;40(15):e115. 22730293 10.1093/nar/gks596 PMC3424584

[43] Zhang Q, Wang J, Deng F, et al. TqPCR: a touchdown qPCR assay with significantly improved detection sensitivity and amplification efficiency of SYBR green qPCR. PLoS One. 2015;10(7):e0132666. 26172450 10.1371/journal.pone.0132666 PMC4501803

[44] Miraglia E, Viarisio D, Riganti C, et al. Na+/H + exchanger activity is increased in doxorubicin-resistant human Colon cancer cells and its modulation modifes the sensitivity of the cells to doxorubicin. Int J Cancer. 2005;115(6):924–929. 15729714 10.1002/ijc.20959

[45] Cleary I, Doherty G, Moran E, et al. The multidrug-resistant human lung tumour cell line, DLKP-A10, expresses novel drug accumulation and sequestration systems. Biochem Pharmacol. 1997;53(10):1493–1502. 9260877 10.1016/s0006-2952(97)00003-8

[46] Inno A, Di Salvatore M, Cenci T, et al. Is there a role for IGF1 R and c-MET pathways in resistance to cetuximab in metastatic colorectal cancer? Clin Colorectal Cancer. 2011;10(4):325–332. 21729677 10.1016/j.clcc.2011.03.028

[47] Shali H, Ahmadi M, Kafil HS, et al. IGF1R and c-met as therapeutic targets for colorectal cancer. Biomed Pharmacother. 2016;82:528–536. 27470393 10.1016/j.biopha.2016.05.034

[48] LeRoith D, Roberts CT. The insulin-like growth factor system and cancer. Cancer Lett. 2003;195(2):127–137. 12767520 10.1016/s0304-3835(03)00159-9

